# The association of early life socioeconomic conditions with prediabetes and type 2 diabetes: results from the Maastricht study

**DOI:** 10.1186/s12939-017-0553-7

**Published:** 2017-04-05

**Authors:** Ivonne P. M. Derks, Annemarie Koster, Miranda T. Schram, Coen D. A. Stehouwer, Pieter C. Dagnelie, Danielle A. I. Groffen, Hans Bosma

**Affiliations:** 1 Department of Social Medicine, Maastricht University, P.O. Box 616, 6200 MD Maastricht, The Netherlands; 2 CAPHRI School for Public Health and Primary Care, Maastricht University, Maastricht, The Netherlands; 3 The Generation R Study Group, Erasmus Medical Centre, Rotterdam, The Netherlands; 4 Department of Child and Adolescent Psychiatry/Psychology, Erasmus MC-Sophia, P.O. Box 2060, 3000 CB Rotterdam, The Netherlands; 5 Department of Medicine, Maastricht University Medical Centre+, Maastricht, The Netherlands; 6 Cardiovascular Research Institute Maastricht (CARIM), Maastricht University, Maastricht, The Netherlands; 7 Department of Epidemiology, Maastricht University, Maastricht, The Netherlands

**Keywords:** Type 2 diabetes, Prediabetes, Childhood socioeconomic conditions, Adulthood socioeconomic conditions, Health behaviour, Obesity

## Abstract

**Background:**

Using cross-sectional data from The Maastricht Study, we examined the association of socioeconomic conditions in early life with prediabetes and T2DM in adulthood. We also examined potential mediating pathways via both adulthood socioeconomic conditions and adult BMI and health behaviours.

**Methods:**

Of the 3263 participants (aged 40–75 years), 493 had prediabetes and 906 were diagnosed with T2DM. By using logistic regression analyses, the associations and possible mediating pathways were examined.

**Results:**

Participants with low early life socioeconomic conditions had a 1.56 times higher odds of prediabetes (95% confidence interval (CI) = 1.21-2.02) and a 1.61 times higher odds of T2DM (95% CI = 1.31-1.99). The relation between low early life socioeconomic conditions and prediabetes was independent of current socioeconomic conditions (OR = 1.38, 95% CI = 1.05-1.80), whereas the relation with T2DM was not independent of current socioeconomic conditions (OR = 1.10, 95% CI = 0.87-1.37). BMI party mediated the association between early life socioeconomic conditions and prediabetes.

**Conclusions:**

Socioeconomic inequalities starting in early life were associated with diabetes-related outcomes in adulthood and suggest the usefulness of early life interventions aimed at tackling these inequalities.

**Electronic supplementary material:**

The online version of this article (doi:10.1186/s12939-017-0553-7) contains supplementary material, which is available to authorized users.

## Background

Type 2 diabetes mellitus (T2DM) has become a worldwide epidemic, with 422 million people having diabetes in 2014 [1]. The prevalence of T2DM shows large inequalities, disproportionally affecting deprived populations [2, 3]. People with low socioeconomic conditions, i.e. as defined by low income, education and occupation, are at higher risk of T2DM, compared to people with higher socioeconomic conditions [2, 4, 5]. However, findings remain inconsistent about the precise influence of socioeconomic conditions early in life and the extent to which such influence acts independent or dependent of adulthood socioeconomic status [3, 6–15]. Knowing that the inequalities already start in early life might also help to better position interventions across the life-course.

Socioeconomic conditions early in life may have lasting effects on different social, biological, and behavioural factors that might act as mechanisms connecting the socioeconomic conditions to later T2DM [16]. Children born in low socioeconomic conditions are more likely to have fewer educational opportunities and therefore maintain their level of socioeconomic conditions in adulthood [17]. Furthermore, children with low socioeconomic backgrounds have a higher risk of developing obesity during childhood [18]. Children often maintain their overweight status through adulthood, which puts them at high risk of developing T2DM later in life [19]. Finally, health behaviours, such as physical inactivity, smoking and alcohol use, might be other mechanisms. For example, low early life socioeconomic conditions have been linked to higher tobacco use and less physical activity in later-life [20–22].

Prediabetes is known as the intermediate state between normal glucose tolerance and T2DM at which people are at high risk for developing T2DM [23, 24]. It is defined as glucose concentrations higher than normal, but lower than the diagnostic thresholds of T2DM and recognizes two states: Impaired Fasting Glucose (IFG) and Impaired Glucose Tolerance (IGT) [23, 25]. The progression towards T2DM can more easily be prevented and the prediabetic state can even be inverted [23, 26–28]. Only one previous study examined the association of early life socioeconomic conditions with prediabetes; the authors reported that they only found evidence for an indirect association [14].

By using cross-sectional data from The Maastricht Study, we aimed to examine whether early life socioeconomic conditions are related to diabetes outcomes in adulthood. This study is the first in the Netherlands that determined the possible influence of socioeconomic inequalities starting early in life on prediabetes and T2DM, and determined the possible pathways via adulthood socioeconomic conditions, adulthood BMI, and adulthood physical activity, smoking, and alcohol use.

## Methods

### Study population

In this study, we used data from The Maastricht Study, an observational prospective population-based cohort study. The rationale and methodology have been described previously [29]. In brief, the study focuses on the etiology, pathophysiology, complications and comorbidities of type 2 diabetes mellitus (T2DM) and is characterized by an extensive phenotyping approach. Eligible for participation were all individuals aged between 40 and 75 years and living in the southern part of the Netherlands. Participants were recruited through mass media campaigns and from the municipal registries and the regional Diabetes Patient Registry via mailings. Recruitment was stratified according to known T2DM status, with an oversampling of individuals with T2DM, for reasons of efficiency.

The present report includes cross-sectional data from the first 3451 participants, who completed the baseline survey between November 2010 and September 2013. The examinations of each participant were performed within a time window of three months. Participants with type 1 diabetes or missing information (*N* = 188) were excluded. This group included more females than the final study population (50.5% versus 48.5%) and this group had a lower educational level (38.2% versus 33.4%). The final study population consisted of 1864 participants without T2DM, 493 participants with prediabetes and 906 participants with T2DM, of whom 130 participants were newly diagnosed on the basis of The Maastricht Study investigations. The study has been approved by the institutional medical ethical committee (NL31329.068.10) and the Minister of Health, Welfare and Sports of the Netherlands (Permit 131088-105234-PG). All participants gave written informed consent.

### Measures

#### Diabetes outcomes

T2DM status was defined according to the WHO diagnostic criteria of glucose tolerance status [25]. All participants underwent a standardized 7-point OGTT after overnight fasting. Blood samples were collected at baseline, and 15, 30, 45, 60, 90 and 120 min after consumption of the 75 g glucose drink. Participants who were insulin-dependent and participants with a fasting glucose level higher than 11.0 mmol/l (as determined by finger prick) did not undergo this test. Prediabetes was defined as IFG (fasting plasma glucose 6.1-6.9 mmol/l and 2-h plasma glucose <7.8 mmol/l), IGT (fasting plasma glucose <7.0 mmol/l and 2-h plasma glucose ≥7.0 - < 11.1 mmol/l) or both [23]. T2DM was defined by fasting plasma glucose ≥7.0 mmol/l or 2-h plasma glucose ≥11.1 mmol/l. Participants on diabetes medication and without type 1 diabetes were also considered as having T2DM.

#### Early life socioeconomic conditions

Early life socioeconomic conditions was measured with poverty in youth and educational level of both parents. Poverty in youth was measured with the following question: “*Was the financial situation at your childhood’s home sometimes such that there wasn’t enough money to buy food or to replace outworn clothes or shoes?”*, with 4 answering categories: “no, never; yes, sometimes; yes, often; yes, always”. Educational level of the parents consisted of eight categories, ranging from no education to university education (i.e. 1. No education, 2. Primary education, 3. Lower vocational education, 4. General secondary education, 5. General vocational education, 6. Higher secondary and pre-university education, 7. Higher vocational education and 8. University). To create an overall index of early life socioeconomic conditions, the three variables were standardized and subsequently averaged. By using tertiles, the scores were categorized into: low, medium and high.

#### Adulthood socioeconomic conditions

Adulthood socioeconomic conditions were measured using income- and educational level of the participant. Income was measured by self-reported net household income per month, consisting of 19 categories, ranging from 0 to >5000 euros per month (sample mean = 3025 per month; SD = 1420.31). To estimate the equivalised household income, the OECD modified scale was used giving weights to the number of persons living in one household with the following calculation: Net household income/1 (participant) + 0.5*no. of extra adults + 0.3*no. of children (<18 years) [30]. Educational level of the participant was measured with eight categories (similar to the ones for early socioeconomic conditions). To compute an overall index of adulthood socioeconomic conditions, the two variables were standardized and averaged. This variable was categorized into tertiles resulting in: low, medium and high.

#### BMI and health behaviours

Participants’ height and weight were measured; BMI was calculated as weight (kg)/height^2^ (m) and categorized according to the WHO [31]. This resulted in three categories: underweight and normal, if BMI <25 kg/m^2^, overweight, if BMI ≥25 kg/m^2^ and <30 and obese, if BMI ≥30 kg/m^2^.

The following health behaviours were included: physical activity, smoking and alcohol use. Physical activity was measured with the CHAMPS questionnaire [32], including sedentary to vigorous activities, and estimated in hours per week. We categorized physical activity into tertiles. This resulted in low physical activity, 0 h to 9.75 h per week, medium physical activity, 9.76 h to 16.25 h per week and high physical activity, 16.26 or more hours per week. Additionally, because there were 399 participants (12.2%) with missing data, a missing category was added. Smoking status was based on self-reported data on smoking cigarettes, cigars and/or pipe tobacco and categorized as: never smokers, former smokers and current smokers. Alcohol use was also based on self-reported data and categorized as: Non-consumers defined as those who did not consume alcohol. Low-consumers were defined as women consuming ≤7 glasses of alcohol per week and men consuming ≤14 glasses of alcohol per week. High consumers were defined as women consuming >7 glasses per week and men >14 glasses a week.

### Statistical analyses

Differences in baseline characteristics by early life socioeconomic conditions were examined with chi-square tests and independent t-tests. Logistic regression analyses were done to study the association of early life socioeconomic conditions with health behaviours and BMI. Likewise, the associations between adulthood socioeconomic conditions, health behaviours, and BMI with prediabetes and T2DM were studied. We separately adjusted for sex and age, and socioeconomic status in adulthood. Subsequently, logistic regression analyses were performed to examine the association between early life socioeconomic conditions, prediabetes and T2DM. This association was first adjusted for age and sex, and secondly also for adulthood socioeconomic conditions (model 1). BMI and the different health behaviours were separately added to the models to study their possible mediating role in the association between early life socioeconomic conditions and both adulthood diabetes outcomes, if model 1 was significant.

Interactions were studied for early life socioeconomic conditions with sex, since some studies found an association between early life socioeconomic conditions and T2DM particularly in females [3, 7, 9, 11]. Moreover, interactions between early life- and adulthood socioeconomic conditions were studied to examine whether specific pathways of social mobility would differ in the association with diabetes outcomes. Analyses were additionally performed in a subsample including only newly diagnosed T2DM participants and participants without diabetes. The newly diagnosed T2DM participants can be expected to be unaware of their diabetes status. This will preclude that their health behaviour, BMI, and adulthood socioeconomic conditions had changed due to any awareness of their disease status. All analyses were performed by using SPSS 21.0.

## Results

Participants brought up in low socioeconomic conditions were more often men (56,7%) compared to participants from high socioeconomic backgrounds (45,6%) (Table 1). Participants with low early life socioeconomic status more often reported low adulthood socioeconomic status than participants with high socioeconomic conditions in early life (49.8% versus 17.0%). Of the participants with low early life socioeconomic conditions, 27.6% were obese, compared to 17.2% of the participants with high early life socioeconomic conditions. Participants with low early life socioeconomic conditions significantly more often had prediabetes or T2DM, compared to participants with high early life socioeconomic conditions.

**Table 1:** Demographics, current socioeconomic conditions, health behaviour and diabetes status by early life socioeconomic conditions, *n* = 3263^a^

		Early life socioeconomic conditions			
		High (*n* = 1051)	Medium (*n* = 1120)	Low (*N* = 1092)	*P*-value
Age		58.33 (8.57)	59.87 (8.14)	60.91 (7.86)	<0.01
Sex	Male	45.6	52.1	56.7	<0.01
	Female	54.4	47.9	43.3	
Current socioeconomic conditions	High	49.8	31.8	20.2	<0.01
	Medium	33.2	36.0	30.1	
	Low	17.0	32.2	49.6	
BMI	Normal	43.0	33.1	29.5	<0.01
	Overweight	39.8	45.2	42.9	
	Obese	17.2	21.7	27.6	
Physical activity	High	30.4	29.3	27.9	0.07
	Medium	28.6	31.0	26.4	
	Low	28.8	30.0	30.9	
	Missing	12.2	9.7	14.8	
Smoking status	Never	35.5	34.6	34.2	0.77
	Former	49.6	53.1	52.2	
	Current	14.9	12.2	13.6	
Alcohol Use	None	15.5	17.2	22.5	<0.01
	Low	54.1	57.7	54.9	
	High	30.4	25.1	22.6	
Diabetes status	No diabetes	64.8	57.9	48.9	<0.01
	Prediabetes	12.5	15.8	16.8	
	T2DM	22.7	26.3	34.2	

^a^Values are percentages, *p*-values of Chi square tests and an independent t-test (age)

As shown in Table 1 already, low socioeconomic conditions in early life are significantly related to similar conditions in adult life. Furthermore, participants with low early life socioeconomic conditions had a 1.83 times higher odds of obesity compared to participants with high early life socioeconomic conditions (95% CI = 1.49-2.25) [Additional file 1]. After adjustment for age, sex and adulthood socioeconomic conditions, this was reduced to 1.27 (95% CI = 1.01-1.85). Other associations were not significant or lost significance when controlled for age, sex and adulthood socioeconomic conditions. Participants with low adulthood socioeconomic conditions had a 1.67 times higher odds of having prediabetes (95% CI = 1.30-2.15) and a 3.43 times higher odds of having T2DM (95% CI = 2.76-4.25) [Additional file 2]. Participants with obesity had a 4.75 times higher odds of prediabetes (95% CI = 3.51-6.41) and a 13.29 times higher odds of T2DM (95% CI = 10.19-17.34). Low physical activity, smoking, and low alcohol use were related to higher odds of both diabetes outcomes, although only statistically significant for the T2DM outcome.

As Table 2 shows, participants with low early life socioeconomic conditions had a 1.56 times higher odds of prediabetes compared with participants with high early life socioeconomic conditions (95% CI = 1.21-2.02). The addition of adulthood socioeconomic conditions results in a reduction of the odds for prediabetes by low early life socioeconomic conditions (OR = 1.38, 95% CI = 1.05-1.80). Controlling for their BMI, the association between low early life socioeconomic conditions and prediabetes was attenuated to 1.35 (95% CI = 1.04-1.76) and 1.26 (95% CI = 0.95-1.66), without and with control for adulthood socioeconomic conditions, respectively. The separate health behaviours hardly contributed.

**Table 2:** Odds ratios for prediabetes and type 2 diabetes versus no diabetes, by early life socioeconomic conditions^a^

Early life socioeconomic conditions		Prediabetes (*n* = 2357)		Type 2 diabetes (*n* = 2770)	
		Adjusted for age and sex Odds ratio (95% CI)	Adjusted for age, sex and current socioeconomic conditions Odds ratio (95% CI)	Adjusted for age and sex Odds ratio (95% CI)	Adjusted for age, sex and current socioeconomic conditions Odds ratio (95% CI)
Model 1	High	1.00	1.00	1.00	1.00
	Medium	1.29 (1.00-1.66)	1.19 (0.92-1.55)	1.10 (0.89-1.36)	0.89 (0.71-1.11)
	Low	1.56 (1.21-2.02)	1.38 (1.05-1.80)	1.61 (1.31-1.99)	1.10 (0.87-1.37)
Model 1, adjusted for BMI	High	1.00	1.00	1.00	1.00
	Medium	1.19 (0.91-1.54)	NA^b^	NA	NA
	Low	1.35 (1.04-1.76)	1.26 (0.95-1.66)	1.31 (1.04-1.65)	NA
Model 1, adjusted for physical activity	High	1.00	1.00	1.00	1.00
	Medium	1.29 (1.00-1.67)	NA	NA	NA
	Low	1.55 (1.20-2.01)	1.37 (1.05-1.79)	1.61 (1.30-1.99)	NA
Model 1, adjusted for smoking status	High	1.00	1.00	1.00	1.00
	Medium	1.30 (1.00-1.68)	NA	NA	NA
	Low	1.57 (1.21-2.03)	1.39 (1.06-1.82)	1.62 (1.31-2.00)	NA
Model 1, adjusted for alcohol use	High	1.00	1.00	1.00	1.00
	Medium	1.29 (1.00-1.67)	NA	NA	NA
	Low	1.54 (1.19-1.99)	1.36 (1.04-1.79)	1.46 (1.17-1.82)	NA

^a^Total *n* = 3263, consisting of no diabetes, *n* = 1864; prediabetes: *n* = 493; T2DM: *n* = 906. ^b^ *NA* not applicable

Table 2 also shows that participants reporting low early life socioeconomic conditions had a 1.61 higher odds of T2DM in later life compared to participants reporting high early life socioeconomic conditions (95% CI = 1.31-1.99). Without control for adulthood socioeconomic conditions, BMI again contributed most; the odds ratio of early life socioeconomic conditions decreased to 1.31 (95% CI = 1.04-1.65). Health behaviours again hardly contributed, except for perhaps alcohol use (OR = 1.46, 95% CI = 1.17-1.82). Inclusion of current socioeconomic conditions attenuated the association to non-significance (OR = 1.10, 95% CI = 0.87-1.37). In a statistical sense, estimating the mediation is then not applicable anymore.

There were no interaction effects between early life socioeconomic conditions and sex, age and adulthood socioeconomic conditions. Sensitivity analyses conducted with newly diagnosed T2DM showed a similar pattern of results as with T2DM. However, including adulthood socioeconomic conditions to the association, did not reduced the odds of T2DM as much: participants with low early life socioeconomic had a 1.93 higher odds of newly diagnosed diabetes in later life compared to participants reporting high early life socioeconomic conditions (95% CI = 1.19-3.12). Adding adulthood socioeconomic conditions in this model, attenuated the association to non-significance (OR = 1.63, 95% CI = 0.98-2.71) [Additional file 3]. Separate inclusion of educational level of the father, mother, and poverty in youth indicated that the combined measure’s influence was not dominated by one socioeconomic measure in particular.

## Discussion

This study examined the association between early life socioeconomic conditions and adulthood diabetes outcomes. Our findings in Dutch middle-aged men and women indicate that adverse socioeconomic conditions in early life are associated with heightened odds of prediabetes and T2DM in adulthood. We further found that such early life conditions increase the odds of both living in similar socioeconomic conditions in adulthood and the odds of obesity in adulthood. Due to the cross-sectional design, it, however, appeared difficult to disentangle the causal directions of the associations between adulthood socioeconomic conditions, BMI, health behaviours, and both diabetes outcomes. Consequently, it appears difficult to be decisive on whether either adulthood or early life socioeconomic conditions are the most important critical period in the aetiology of diabetes. Evidence is thus most solid and robust for the long-term influence of early life socioeconomic conditions on adulthood diabetes and the possible mechanisms via later socioeconomic pathways (stability) and obesity development. Health behaviours appear to contribute only little to the association in this sample.

Given the absence of longitudinal information on the association between current socioeconomic status and odds of diabetes, it is impossible to definitively confirm or falsify any of the different life-course models. Although adulthood socioeconomic conditions attenuated the association between early life socioeconomic conditions and T2DM, this cannot be interpreted as adulthood socioeconomic conditions are a more critical period. It might be possible that socioeconomic conditions changed due to the disease [33]. When only people were analysed who were not aware of their T2DM status, adulthood socioeconomic conditions attenuated the early life effect less. It is not clear whether and to what extent this can be interpreted as that the awareness of T2DM indeed caused people to be downwardly mobile in socioeconomic terms. Hence, we have to wait for longitudinal data to better study the different life-course models in our study. As a final note, no interaction effect was found between early life socioeconomic conditions and adulthood socioeconomic conditions, indicating that different socioeconomic pathways (e.g. being brought up in good socioeconomic conditions and going down in socioeconomic conditions during adulthood) in this sample are not likely to specifically add to the “prediction” of diabetes outcomes [34].

Given the relative stability of inequalities in socioeconomic conditions during the life course (only 20.2% of low early life socioeconomic conditions reached high adulthood socioeconomic conditions compared to 49.8% of high early life socioeconomic conditions) and the association with obesity and both diabetes outcomes, our findings foremost indicate the importance of early life socioeconomic conditions for both socioeconomic and diabetes-related life-course pathways. Previous studies have confirmed the importance of early life socioeconomic conditions for T2DM [13], whether it was only via adulthood socioeconomic conditions [3, 6, 8, 10, 14] or also independently [7, 9, 11, 12, 15]. Some of these studies confirm the pathway via (abdominal) obesity [3, 9, 12, 14, 15]. In other studies, the relationship between early life socioeconomic conditions and T2DM was particularly found in women [3, 7, 9, 11]. We could not confirm this in our sample, the interaction with sex was not statistically significant.

Why might the pathway via obesity be so important? First, low maternal socioeconomic conditions are related to lower birthweight; this in turn might be related to a higher risk of obesity and T2DM [35, 36]. Second, parents in lower socioeconomic conditions may have less supportive interactions with their children and less authority in parenting [37]; this might also shape children’s lifestyles, including eating habits and physical exercise [37, 38]. Given that physical activity did hardly contribute to the relevant associations, it might be worthwhile to specifically study socioeconomic patterns in eating habits. Third, higher stress levels in lower early life socioeconomic conditions might activate inflammation processes [6, 39, 40], including ones related to obesity and T2DM risk [6, 41]. Fourth, another psychosocial pathway might be via low perceived control, which has been found more common in lower socioeconomic circumstances, also those in early life [42, 43]. Low control beliefs can in turn influence people’s health behaviours [44], e.g. by increased beliefs of being unable to lose weight.

This study has several limitations. As said above, the cross-sectional design excludes the possibility of testing for the causal direction of associations; results should thus be interpreted with caution, especially for the T2DM outcome. Furthermore, recall bias and, particularly for the T2DM patients, social desirability might have affected our findings regarding health behaviours, but this bias might be less problematic for the reports on the socioeconomic conditions [45]. Finally, participants with T2DM in our study are relatively young with generally well-regulated type 2 diabetes. It is uncertain how this all might have affected our findings.

## Conclusion

This study shows that socioeconomic conditions early in life are associated with prediabetes and T2DM in adulthood. Although there is a strong need for longitudinal confirmation, adult obesity and low socioeconomic status in adulthood appear to be important pathways, more so than pathways via health behaviours. To address T2DM inequalities, interventions should thus consider improving socioeconomic conditions already in childhood.

## Additional files

Additional file 1: Table S1. Associations between early life socioeconomic conditions and health behaviour and BMI, *n* = 3263. (PDF 146 kb)

Additional file 2: Associations between BMI, health behaviour and current socioeconomic conditions with diabetes status. (PDF 145 kb)

Additional file 3: Table S3. Odds ratios for newly diagnosed diabetes by early life socioeconomic conditions. (PDF 146 kb)
