# Supplementary material for: The association of early life socioeconomic conditions with prediabetes and type 2 diabetes: results from the Maastricht study
Source: Int J Equity Health. 2017 Apr 5;16:61. doi: 10.1186/s12939-017-0553-7 (PMC5382485; doi:10.1186/s12939-017-0553-7)
Supplement: Supplementary file 1 — Associations between early life socioeconomic conditions and health behaviour and BMI, n = 3263. (PDF 146 kb) [file 12939_2017_553_MOESM1_ESM.pdf]

Supplement table 1. Associations between early life socioeconomic conditions and health behaviour and BMI, n=3263[a]

			Health behaviour		
Early life socioeconomic conditions		BMI (>30) Odds Ratio (95% CI)	Physical Activity (Low) Odds Ratio (95% CI)	Smoking Status (Current smoker) Odds Ratio (95% CI)	Alcohol use (High) Odds Ratio (95% CI)
Model 1, unadjusted	High	1.00	1.00	1.00	1.00
	Medium	1.33 (1.08-1.65)	1.06 (0.88-1.27)	0.79 (0.62-1.02)	0.77 (0.64-0.93)
	Low	1.83 (1.49-2.25)	1.10 (0.92-1.33)	0.90 (0.71-1.15)	0.67 (0.55-0.81)
Model 2, adjusted for sex and age	High	1.00	1.00	1.00	1.00
	Medium	1.30 (1.05-1.61)	1.02 (0.84-1.23)	0.84 (0.65-1.07)	0.75 (0.62-0.90)
	Low	1.76 (1.42-2.17)	1.03 (0.85-1.25)	0.98 (0.77-1.26)	0.64 (0.52-0.78)
Model 3, adjusted for sex, age and current socioeconomic conditions	High	1.00	1.00	1.00	1.00
	Medium	1.09 (0.87-1.36)	1.00 (0.82-1.21)	0.73 (0.57-0.95)	0.87 (0.71-1.06)
	Low	1.27 (1.01-1.58)	0.99 (0.81-1.21)	0.78 (0.60-1.01)	0.85 (0.69-1.05)

[a] Outcome measures were dichotomized by comparing the worst category to the other two categories combined.
