# Supplementary material for: The association of early life socioeconomic conditions with prediabetes and type 2 diabetes: results from the Maastricht study
Source: Int J Equity Health. 2017 Apr 5;16:61. doi: 10.1186/s12939-017-0553-7 (PMC5382485; doi:10.1186/s12939-017-0553-7)
Supplement: Supplementary file 2 — Associations between BMI, health behaviour and current socioeconomic conditions with diabetes status. (PDF 145 kb) [file 12939_2017_553_MOESM2_ESM.pdf]

Supplement table 2.

Associations between BMI, health behaviour and current socioeconomic conditions with diabetes status [a]

		Prediabetes (total n=2357) Odds ratio (95% CI)	T2DM (total n=2770) Odds ratio (95% CI)
Current socioeconomic conditions	High	1.00	1.00
	Medium	1.33 (1.04-1.71)	1.69 (1.35-2.11)
	Low	1.67 (1.30-2.15)	3.43 (2.76-4.25)
BMI	Normal	1.00	1.00
	Overweight	2.23 (1.75- 2.85)	2.87 (2.28- 3.62)
	Obese	4.75 (3.51- 6.41)	13.29 (10.19-17.34)
Physical activity	High	1.00	1.00
	Medium	0.98 (0.75- 1.28)	1.16 (0.92-1.47)
	Low	1.27 (0.97- 1.66)	1.88 (1.50-2.37)
Smoking status	Never	1.00	1.00
	Former	1.39 (1.11- 1.75)	1.27 (1.05-1.54)
	Current	1.39 (0.99- 1.95)	1.77 (1.35-2.31)
Alcohol use	None	1.00	1.00
	Low	0.65 (0.48- 0.88)	0.26 (0.20- 0.32)
	High	0.75 (0.55- 1.04)	0.21 (0.16- 0.27)

[a] All models were adjusted for age and sex.
