# Supplementary material for: The association of early life socioeconomic conditions with prediabetes and type 2 diabetes: results from the Maastricht study
Source: Int J Equity Health. 2017 Apr 5;16:61. doi: 10.1186/s12939-017-0553-7 (PMC5382485; doi:10.1186/s12939-017-0553-7)
Supplement: Supplementary file 3 — Odds ratios for newly diagnosed diabetes by early life socioeconomic conditions. (PDF 146 kb) [file 12939_2017_553_MOESM3_ESM.pdf]

Supplement Table 3. Odds ratios for newly diagnosed diabetes by early life socioeconomic conditions.[a]

	Early life socioeconomic conditions	Newly diagnosed diabetes (n=1994) Adjusted for age and sex Odds ratio (95% CI)	Adjusted for age, sex and current socioeconomic conditions Odds ratio (95% CI)
Model 1	High	1.00	1.00
	Medium	1.59 (0.98-2.58)	1.45 (0.89-2.37)
	Low	1.93 (1.19-3.12)	1.63 (0.98-2.71)
Model 1, adjusted for BMI	High	1.00	1.00
	Medium	NA [b]	NA
	Low	1.52 (0.92-2.50)	NA
Model 1, adjusted for physical activity	High	1.00	1.00
	Medium	NA	NA
	Low	1.90 (1.17-3.09)	NA
Model 1, adjusted for smoking status	High	1.00	1.00
	Medium	NA	NA
	Low	1.94 (1.20-3.14)	NA
Model 1, adjusted for alcohol use	High	1.00	1.00
	Medium	NA	NA
	Low	1.89 (1.16-3.07)	NA

[a] No diabetes, n=1864; newly diagnosed diabetes: n=130. [b] NA: not applicable.
